# 

**Published:** 1902-11-15

**Authors:** 


					yr ibe hospital "The standard of highest purity ?Lancet. Nov. 15th. 1902,
CADMRY'? ^ u ** CADBURY'S
COCOA
ABSOLUTELY PURB.
OOOOA
MO DRUGS OR ALKALIES U??
which uasBijAA
Ko. 842. Vol. XXXIII. [aSpfpen]
Saturday, Nov. 15th, 1902.
L SeePpTo8 J PRICB TWOPENCE

				

